# Interstitial Cells of Cajal and Enteric Nervous System in Gastrointestinal and Neurological Pathology, Relation to Oxidative Stress

**DOI:** 10.3390/cimb45040232

**Published:** 2023-04-18

**Authors:** Laura López-Pingarrón, Henrique Almeida, Marisol Soria-Aznar, Marcos C. Reyes-Gonzales, Ana B. Rodríguez-Moratinos, Antonio Muñoz-Hoyos, Joaquín J. García

**Affiliations:** 1 Department of Pharmacology, Physiology and Legal and Forensic Medicine, Faculty of Medicine, University of Zaragoza, 50009 Zaragoza, Spain; msoria@unizar.es (M.S.-A.); mreyesg@unizar.es (M.C.R.-G.); jjgarcia@unizar.es (J.J.G.); 2 i3S—Instituto de Investigação e Inovação em Saúde, Porto University, 4200-135 Porto, Portugal; almeidah@med.up.pt; 3 Department of Biomedicine, Faculty of Medicine, Porto University, 4200-319 Porto, Portugal; 4 Department of Obstetrics and Gynecology, Hospital-CUF Porto, 4100-180 Porto, Portugal; 5 Department of Physiology, Faculty of Science, University of Badajoz, 06006 Badajoz, Spain; moratino@unex.es; 6 Department of Pediatrics, Faculty of Medicine, University of Granada, 18016 Granada, Spain; amunozh@ugr.es

**Keywords:** enteric nervous system, interstitial cells of Cajal, oxidative stress, neurodegeneration, myenteric plexus

## Abstract

The enteric nervous system (ENS) is organized into two plexuses—submucosal and myenteric—which regulate smooth muscle contraction, secretion, and blood flow along the gastrointestinal tract under the influence of the rest of the autonomic nervous system (ANS). Interstitial cells of Cajal (ICCs) are mainly located in the submucosa between the two muscle layers and at the intramuscular level. They communicate with neurons of the enteric nerve plexuses and smooth muscle fibers and generate slow waves that contribute to the control of gastrointestinal motility. They are also involved in enteric neurotransmission and exhibit mechanoreceptor activity. A close relationship appears to exist between oxidative stress and gastrointestinal diseases, in which ICCs can play a prominent role. Thus, gastrointestinal motility disorders in patients with neurological diseases may have a common ENS and central nervous system (CNS) nexus. In fact, the deleterious effects of free radicals could affect the fine interactions between ICCs and the ENS, as well as between the ENS and the CNS. In this review, we discuss possible disturbances in enteric neurotransmission and ICC function that may cause anomalous motility in the gut.

## 1. Introduction

**Figure 1:**
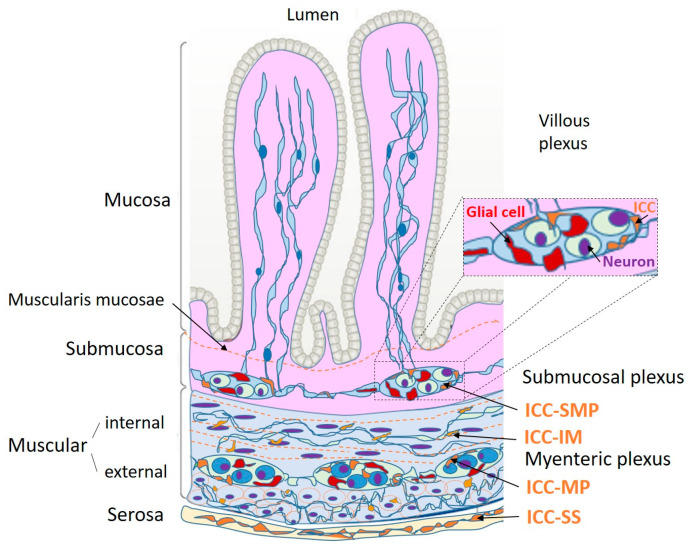
Organization of interstitial cells of Cajal (ICC) at submucosal and myenteric plexuses in the digestive tube. There are three principal cells represented in plexuses: neurons, glial cells, and the ICCs. Some prolongations end into mucosa. ICC-SMP: ICC of submucosal plexus; ICC-MP: ICC of myenteric plexus; ICC-IM: ICC intramuscular at internal and external muscular; ICC-SS: ICC of serosa. Modified from [5].

The wall of the digestive system is endowed with a complex neuronal network known as the enteric nervous system (ENS). The ENS is responsible for gastrointestinal motility patterns; adequate digestive secretion of different electrolytes, enzymes, and mucus, depending on the anato-mophysiological region; and orchestrating the immune and defensive functions of the digestive tract [1]. The ENS is distributed throughout the digestive tract in two plexuses (Figure 1): the myenteric (or Auerbach’s) plexus, which forms ganglia that are recognized between the longitudinal and circular muscle layer, and the submucosal (or Meissner’s) plexus, which is located between the mucosa and the inner muscular layer in the submucosa [2,3]. The myenteric plexus is primarily responsible for the control of muscle contraction, whereas the submucosal plexus controls secretory functions and blood flow regulation [4].

Embryologically, the ENS is derived from neural crest cells, mostly of vagal origin, which migrate through the paraxial mesoderm to the digestive tract. From this point, they migrate in a rostrocaudal direction and end up colonizing the entire intestinal tract [4]. Immuno-histochemical studies have shown that the axons of excitatory motor neurons project cranially, whereas inhibitory neurons project caudally [3,4].

Although the ENS acts by itself independently of the rest of the autonomic nervous system (ANS), the stimulations from the sympathetic and parasympathetic systems enhance or attenuate its specific actions, influencing gastrointestinal activity. It is estimated to be made up of approximately 100 million neurons, which is at least as many as the spinal cord [1,6]. It has come to be known as “the second brain”, even postulating that it has learning and memory capacity [6]. Its neurotransmission agents are extensive and continue to be investigated. Catecholamines, especially norepinephrine, are considered to be the main neurotransmitters of ANS sympathetic postganglionic fibers [7]. Enterochromaffin cells of the intestinal mucosa synthesize 95% of serotonin (5-HT) in humans from dietary tryptophan [7]. Myenteric neurons can also produce serotonin [7]. Their importance as a paracrine mediator in the gastrointestinal tract has been highlighted previously [8]; they may be both a pro-inflammatory and anti-inflammatory factor at the intestinal level [7]. In this sense, the neuronal plasticity of the ENS has been studied in animal models, especially after inflammatory episodes, which indicate that changes induced by inflammation promote synaptic rearrangement and changes in motility [6].

Although a mutual relationship exists between the ENS and central nervous system (CNS), the former seems to function more as a receiver of information, considering that up to 90% of the fibers of the vagus nerve are of an afferent nature [1]. Stimuli received by the CNS can initiate vagal reflexes in response to enteric stimuli to regulate gastrointestinal motility patterns. For example, this enteric–cerebral signaling regulates and manifests the sensations of satiety and nausea. The role of the CNS in its efferent connections to the gastrointestinal tract is noticeable in the most proximal and distal sections of the digestive tract as it is essential in chewing, swallowing, and defecation. [1]. However, the ENS is in charge of controlling locally the functions of the small intestine and colon; in fact, even if the CNS is disconnected from the ENS, the basic enteric motility is maintained [6]. Vagal reflexes also control gastric motility, allowing its capacity to be increased without increasing intraluminal pressure, which is essential for the content to be catabolized and transported to the duodenum [1].

Despite the independence of the ENS from the CNS, the functioning of the gut requires integrity of the ENS along its extension as an affection in a small section is likely to result in serious consequences to general intestinal function. This can manifest as a pseudo-obstruction due to congenital or acquired diseases, such as Hirschsprung’s or Chagas disease, respectively [1], or may have iatrogenic causes as in the case of post-surgical ileus [9]. Hormonal signals, such as adrenaline, oxytocin, or corticotropin, can influence the activity of the ENS, which may partly explain the close relationship between anxious states and digestive disorders [3]. Neurotransmission in the ENS is similar in complexity to that in the brain as the amount and type of neurotransmitters found are the same [1]. Roughly 20 neuronal subtypes have been described according to neurotransmission; their modification and differentiation depend on both genetic and environmental factors [4]. The ENS also exerts its functions by interacting with the immune and epithelial cells of the intestinal lumen [3]. For example, the neurons of the myenteric plexus have an important relationship with the macrophages of the *muscularis mucosae* [3,10]. The ENS may also stimulate mast cells, which are mainly located in the lamina propria of the intestine, epithelium, submucosal layer, and serosa [10], to release the vasodilator histamine directly onto submucosal blood vessels via substance P and/or calcitonin gene-related peptide (CGRP) [3]. ENS interacts, through gap junctions, with a multicellular syncytium, formed by smooth muscle cells, ICCs, and fibroblast-like platelet-derived growth factor receptor (PDGFR)+ cells, being critical for gut motility [3].

Different neurological conditions, especially neurodegenerative disorders, have relevant manifestations at the gastrointestinal level. Some examples of neurological diseases that affect both the CNS and ENS are transmissible spongiform encephalopathies, Parkinson’s disease (PD), Alzheimer’s disease (AD), amyotrophic lateral sclerosis (ALS), varicella zoster virus (VZV) infection, and autism spectrum disorders (ASD) [1]. ENS dysfunction has also been linked to irritable bowel syndrome, inflammatory bowel disease (IBD), and gastroparesis [4]. An increase in neuronal density in intestinal biopsies from patients with irritable bowel syndrome, and in patients complaining of chronic abdominal pain, has been shown [6].

## 2. The Interstitial Cells of Cajal in the Gut

The interstitial cells of Cajal (ICCs) are pacemaker cells of gastrointestinal function that control motility and communicate with enterocytes, blood vessels, smooth muscle cells, and enteric plexus neurons [3,11]. The ICCs take the name of the father of modern neuroanatomy, Santiago Ramón y Cajal, because he described them for the first time [12].

During their migration towards the digestive tube, simultaneously with the neural crest cell reaching the gut and starting to differentiate into enteric neurons and glia, ICCs and muscles differentiate from the mesenchymal lineage [13]. This process has been demonstrated in vitro through the induction of pluripotent cells combined with cells derived from the neural crest, which migrate to the mesenchyme and form neuroglial structures and myenteric plexuses with functional ICCs [14]. For this reason, though in the past ICCs were recognized as a type of neuron, they are currently considered to be mesenchymal cells [15,16]. However, the ICCs of the circular muscle layer may derive from the ventral zone of the neural tube (VENT cells) [17] according to a study in which they were identified in the stomach and duodenum of chicken embryos [18].

**Figure 2:**
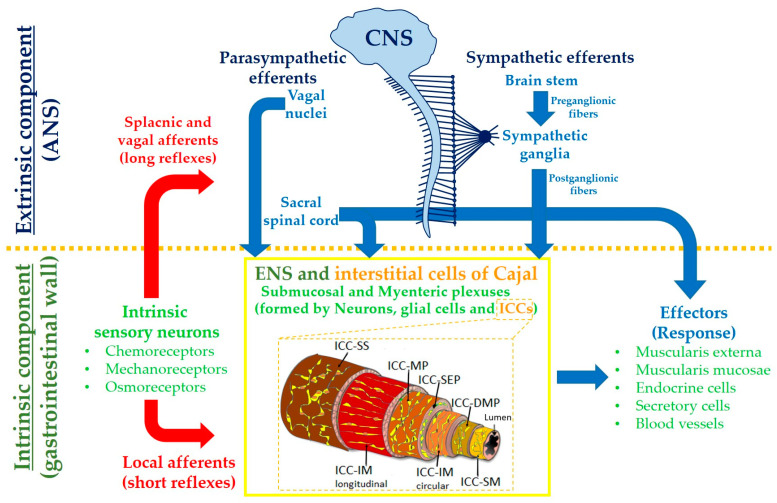
Representation of link between central nervous system (CNS), autonomic nervous system (ANS) and enteric nervous system (ENS). Location of interstitial cells of Cajal (ICCs) sub-types according to the gastrointestinal layer. ICC-SM: ICC of submucosa; ICC-DMP: ICC of deep muscle plexus; ICC-IM: ICC of intramuscular layer; ICC-MP: ICC of myenteric plexus; ICC-SS: ICC of sub-serosa. Modified from [11].

ICCs can be divided according to their histological location. Thus, ICC-SM correspond to those found in the submucosa [19] and are present in the stomach. ICC-SMP are found in the colon [10]. ICC-MP correspond to those of the myenteric or Auerbach plexus. The intramuscular ICCs, known as ICC-IM, are further divided into ICC-CM and ICC-LM according to their localization in the circular or longitudinal layers [10] (Figure 2).

ICCs of the esophagus are of the ICC-IM type and found along the esophageal smooth muscle and lower esophageal sphincter [19,20]. Similar cells appear in the colon and stomach, either in groups or isolated [10]. In the stomach, the ICCs are more frequent in the body and antrum, especially in the pyloric sphincter, where both ICC-IM and ICC-SM sub-types are found [19]. ICC-MP also appear in the aforementioned locations but are absent at the fundus level [10]. In the small intestine, they are intimately associated with the myenteric plexus, where they predominate [10] and have receptors for neurotransmitters and hormones, such as cholecystokinin (CCK) [19] and CCK-A receptors, involved in digestion and gallbladder contraction [21]. ICC-MP density is significantly higher in the ileum than in other divisions [19]. In the colon, the subtypes are ICC-MP, ICC-IM, and ICC-SM and exhibit a lower density in the internal anal sphincter [19]. The presence of ICCs has also been reported in the vermiform appendix [22].

Notably, ICCs are not exclusively located in the gut wall. They have been demonstrated in other organs, such as the pancreas, bladder, myometrium, myocardium, urethra, fallopian tubes, uterus, vagina, and human placenta [19,20,22]. In his early observations, Ramón y Cajal interpreted ICCs as primitive neurons, though their numerous branches challenged his theory of neurons as independent units [20]. However, because immuno-histochemical techniques had not yet been developed, the stains used for visualization under the light microscope, such as methylene blue or the Golgi method using silver impregnations, did not provide reliable information for their identification [20].

ICCs are described as isolated or small groups of cells with long processes and varicosities that extend between septa separating the smooth muscle fibers [20]. In studies on ICCs focused on the rodent gastrointestinal tract, they were present throughout the gut in groups of morphologically common cells. Sub-serosa ICCs (ICC-SS) exhibited a stellate morphology, whereas ICC-MP belonging to Auerbach’s plexus formed networks around myenteric ganglia with multipolar morphology [20]; ICC of the deep muscular plexus (ICC-DMP) had bipolar structures in the deep muscular layers of the small intestine [10].

It was not until the development of electron microscopy that the internal structure of ICCs was evidenced [20]. Comparative morphology studies by Komuro et al. in 1999 served as the basis for the cytological definition of ICCs as separate and heterogeneous cellular entities according to tissue type [23,24]. In general, the main features include a large number of mitochondria, intermediate filaments, and gap junctions [24], as well as a large nucleus compared to the cytoplasm [22].

**Figure 3:**
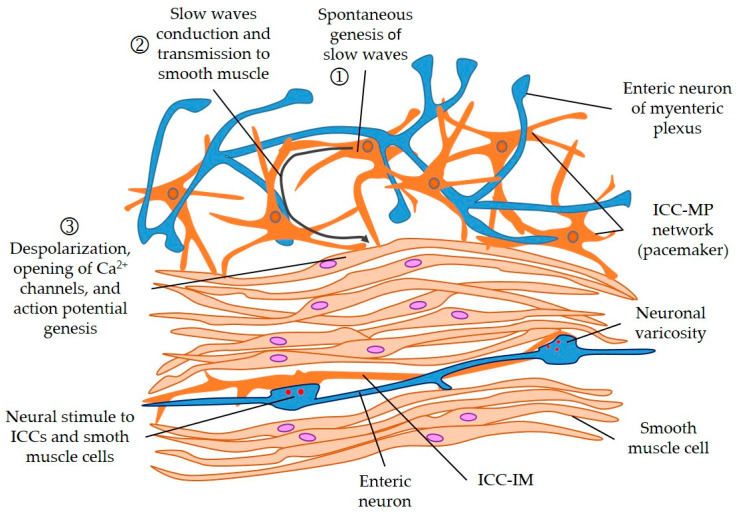
Genesis and conduction of slow waves produced by interstitial cells of Cajal (ICCs) and its transmission to smooth muscle cells. Smooth muscle cells and ICC receive input from the varicosities of enteric neurons (in blue color). ICC-MP: ICC of myenteric plexus; ICC-IM: ICC intramuscular. Modified from [23].

Rodent ICC-CM have a very electron-dense cytoplasm, ovoid nuclei, dense heterochromatin at the periphery, numerous mitochondria [24], and abundant intermediate filaments, mostly in cytoplasmic processes that extend in various directions [24]. They stand out in the number of caveolae they have and the close contact they establish with the neuronal synaptic endings containing the synaptic vesicles. Their most distinctive feature is the reticular organization, forming connections with smooth muscle cells through gap junctions [23] (See Figure 3). Similar interstitial cells can be identified around muscular locations, which is reminiscent of fibroblasts. Their distinguishing features are a moderately electron-dense cytoplasm, well-developed endoplasmic reticulum and Golgi apparatus, and less notable gap junctions with neighboring smooth muscle myocytes [24].

The ICCs of myenteric plexuses employ gap junctions to connect with neurons of the enteric nervous system and smooth muscle cells [20], as well as with the outermost part of the circular muscle layer [10]. The ICC-MP have a triangular or spindle cell morphology, with an elongated nucleus and two to five cytoplasmic processes branching around them in new dendritic processes [22]. However, the ICC-DMP seem to form less compact groups and have more rounded structures, though they maintain the cytoplasmic extensions parallel to the circular muscle layer [24]. The ultrastructure of these ICCs exhibits strong similarity to smooth muscle cells, with numerous caveolae and a distinguishable basal lamina [24].

Both ICCs and mast cells depend on stem cell factor (SCF), or Steel, for their development; both also overexpress the proto-oncogene *c-kit*, which encodes the KIT tyrosine kinase receptor [10,20]. The identification of this membrane receptor has made it possible to study the distribution of Cajal cells through immuno-histochemical methods, using the CD117 protein as a marker [10,20]. Mast cells, which also stain positive for this marker, are capable of secreting important mediators for the development of ICCs, such as SCF or IL-9 [10]. In addition, the expression of the *ANO1* gene product was identified in ICCs from KIT+ gastrointestinal stromal tumors (GISTs) and the human gastrointestinal tract [11]. *ANO1* encodes calcium-activated chloride channels (CaCCs) [11] and can serve as a selective molecular marker for ICCs [16]. Up to four different variants of *ANO1* (a, b, c, and d) have been described, with *b* being more sensitive to calcium [11].

Another marker that has been studied is CD34 as some ICCs express it [25], though there is still controversy about this because CD34 is also expressed by fibroblast-like cells. These cells are positive for platelet growth factor receptor alpha (PDGFRα) but are c-kit negative, which differentiates them from ICCs [26]. On the other hand, in contrast to neighboring smooth muscle myocytes, ICCs react to vimentin [22]. They have also been related to the *WT-1* gene involved in Wilms tumor, as well as calretinin [20].

Neurons are one of the sources of SCF [20], suggesting that they play a prominent role in the differentiation of precursor c-kit cells. The possible role of these cells in the migration and differentiation of neural crest cells into neurons, glial cells, and myenteric ganglia formation, as well as in the differentiation of smooth muscle cells apart from ICCs, has been proposed [13].

One of the main functions of ICCs is to be a pacemaker cell [10]. In electro-physiological studies of the gastrointestinal tract that have shown peristaltic activity, ICCs have been found to be involved [11,21]. Specifically, ICC-SM generate slow waves that propagate the contractions but do not propel them, whereas the ICC-MP of Auerbach’s plexus would generate stimulus-dependent propulsive motor complexes [27].

These slow waves appear to be generated independently by ICCs rather than smooth muscle myocytes, which were first thought to be their originators [22]. It has been possible to implant ICCs in myenteric tissues that lack them, resulting in the generation of slow waves and peristaltic function [28]. The mechanism that explains the pacemaker activity of ICCs is still under study. These cells have been shown to form networks both among themselves and with smooth muscle cells and cells expressing platelet growth factor receptors, such as PDGFRα [11].

Another important function of ICCs is their ability to perform enteric neurotransmission. Apart from the KIT signaling pathway, new mediators of ICC differentiation and proliferation have been described, such as neuronal nitric oxide (NO), the serotonergic receptor 5-HT2B, insulin, and the growth factor IGF-1, as well as immunomodulators such as interleukin-9 [13]. In addition, ICCs have receptors for neuropeptides, such as substance P or neurokinin K and sensitivity to NO [4,21]. Another study demonstrated the presence of the enzyme heme oxygenase 2 (HO-2) in human pyloric ICC-IM; thus, these intramuscular ICCs were proposed to endogenously synthesize carbon monoxide (CO) through HO-2 as another molecular messenger with smooth muscle [29].

A third function attributed to ICCs is their mechanoreceptor activity, which is responsible for the transduction of a mechanical stimulus into an electrical signal that causes motility of the digestive tract [30]. A new sub-type of ICCs was described in the muscular septa, known as the ICC-SEP, the main function of which is to be a mechanoreceptor [13]. The basis of this mechanosensitivity has been suggested to be in the various transmembrane ion channels of the ICCs, which are mainly calcium, sodium, and potassium [30].

The intramuscular formations of the vagus nerve (IMF) along the digestive tract are considered to be the main mechanoreceptor structures of the digestive system. By means of electron microscopy of the mouse gastric fundus, a close communication between the IMF and ICC-IM has been evidenced; this connection has been described as presynaptic [31].

## 3. Oxidative Stress in the Interstitial Cells of Cajal and the Enteric Nervous System

**Figure 4:**
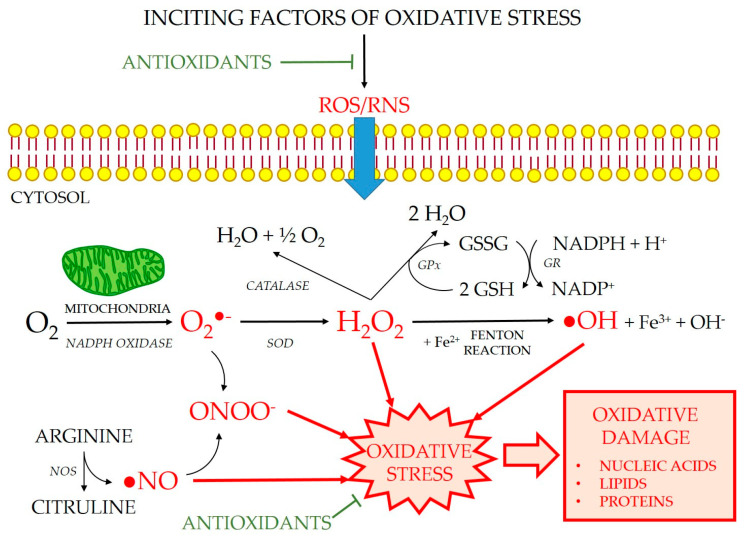
Generation of reactive oxygen and nitrogen species (ROS and RNS). ROS are produced during the normal aerobic cell metabolism. Superoxide anion (O_2_^•−^) is formed from oxygen (O_2_) mainly in the mitochondria. O_2_^•−^ either reacts rapidly with nitric oxide (^•^NO) to produce toxic peroxynitrite (ONOO^−^) or is catalyzed by superoxide dismutase (SOD) to generate hydrogen peroxide (H_2_O_2_), which can be neutralized by catalase or glutathione peroxidase (GPx). In presence of transition metal ions, such as ferrous ion, H_2_O_2_ forms hydroxyl radicals (^•^OH) by the Fenton reaction. GR: glutathione reductase; GSH: reduced glutathione; GSSG: oxidized glutathione; NADP: nicotinamide adenine dinucleotide phosphate; NADPH: reduced NADP; NOS: nitric oxide synthase. Modified from [40].

A free radical (FR) is defined as any chemical species, atom, molecule, or part thereof capable of independent existence that possesses one or more unpaired electrons (e-) in an atomic orbital [32,33]. This circumstance gives them a very unstable electronic configuration [34,35]. These include superoxide (O_2_^•−^), hydroxyl (^•^OH), alkoxyl (RO^•^), and peroxyl (ROO^•^) radicals. The concept of reactive oxygen and nitrogen-dependent species (ROS, RNS) includes all FR and those molecules that, without being a FR, generate FR or are formed in their metabolism. This category includes singlet oxygen (^1^O_2_), hydrogen peroxide (H_2_O_2_), hypochlorous acid (HClO), and peroxynitrite anion (ONOO^−^). In the eukaryotic cells of living organisms, the main endogenous sources of FR and ROS are the mitochondria, which exist in their electron transport chain and during fatty acid metabolism [36,37]. They are also formed in reactions catalyzed by peroxisome cytochromes, the respiratory burst during phagocytosis, involving enzymes such as NADPH oxidase, which forms O_2_^•−^ [37,38], and the nitric oxide synthase (NOS) which produces nitric oxide (NO) from L-arginine and oxygen [39] (Figure 4).

An antioxidant can be defined as any substance that, at low concentrations compared to the oxidizable substrate, significantly decreases or inhibits the oxidation of this substrate [41,42]. Therefore, an antioxidant is any molecule capable of combating and reducing the toxicity of FR. From a functional point of view, antioxidants are classified into enzymatic and non-enzymatic. Enzymatic antioxidants are high molecular weight proteins that minimize oxidative damage by catalyzing chemical reactions, whereas non-enzymatic antioxidants are small molecules that react directly with FR [43]. The major antioxidant enzymes include superoxide dismutase (SOD), catalase (CAT), and glutathione peroxidase (GPx) and its coupled enzyme for maintaining glutathione homeostasis, glutathione reductase (GR). It also includes heme oxygenase (HO), which catalyzes the metabolism of the heme group and biliverdin, ferrous iron, and carbon monoxide. The group of non-enzymatic antioxidants is very broad and includes glutathione, several vitamins, melatonin, uric acid, bilirubin, chelators, and flavonoids, among others.

Oxidative stress (Figure 4) is defined as an imbalance between pro- and anti-oxidant molecules due to an excess of pro-oxidants (by exposure to ionizing radiation, intense ultraviolet radiation, toxins, psychological or physical stress, and diseases), antioxidant deficiency (in newborns, during aging, or due to vitamin-poor diets), or by a combination of both situations at the same time [44]. The action of FR on the cellular structural constituents produces a continuous internal aggression, which threatens the integrity of all biomolecules. Oxidizable substrates include all organic or inorganic molecules found in living cells, such as proteins, lipids, carbohydrates, and deoxyribonucleic acid molecules [45]. Oxidative damage to lipids, especially polyunsaturated fatty acids in biological membranes, is widely investigated [46]. The lipid peroxidation reaction is a degenerative process of chain oxidations that spreads across the surface of lipid bilayers and alters their fluidity and permeability, favoring the occurrence of chemotaxis and inflammation [47,48].

Therefore, oxidative stress is a biochemical concept that can be summarized as a state of imbalance between oxidizing and antioxidant substances that favors the former [49]. There appears to be a close relationship between oxidative stress and gastrointestinal dysfunction; however, oxidative stress also has beneficial involvement, such as coping with infectious processes, and is only harmful when there is a loss of that balance [49].

Of the myriad enzymes involved in oxidative stress, the present review focuses specifically on nitric oxide synthase (NOS), heme oxygenase (HO), and superoxide dismutase (SOD). One of the most studied enzymes in the ENS is NOS, which forms NO from the amino acid L-arginine [50]. NO is an inhibitory neurotransmitter produced by enteric neurons and activates the enzyme NO guanylate cyclase (NO-GC) [51], which is present in brain and intestinal tissue. At the ENS level, the presence of NO-GC has been demonstrated at the myenteric plexus, as well as in smooth muscle cells and the ICCs [52].

In jejunum samples from mice exposed to pro-inflammatory cytokines, interferon-gamma-lipopolysaccharide (IFNγ-LPS)-mediated inflammation was found to affect the pacemaker function of ICCs, though there were no ultrastructural or apoptotic changes. In addition, the phenotypic changes in ICCs produced by the inflammatory state were due to oxidative stress induced by the NO synthesized by macrophages and smooth muscle cells [53]. In patients with neurodegenerative diseases, such as AD and ALS, NADPH oxidase 2 (NOX2) in serum is increased compared to control patients, suggesting that NOX2 enzyme activity is significantly increased in these cohorts. Moreover, plasma levels of LPS, derived from intestinal Gram-negative bacteria, are significantly increased in AD, PD, and ALS patients [54]. Finally, in states of sepsis, the release of interleukin 17 (IL-17) activates macrophages in the muscle layer, which in turn structurally damage the ICCs due to NOS-induced oxidative stress [55].

The synthesis of NO in ICC-MP and ICC-SM has been proposed to enhance intracellular calcium, suggesting a role in the amplification and propagation of inhibitory signals [56]. When this intracellular calcium is abnormally elevated, free radicals are produced in the cytoplasm, which increases damage [57]. Although there is no experimental evidence in ICCs, NOS overexpression in neurons has been linked to several enteropathies, most notably gastroparesis with loss of nitrergic neuron functions, as well as achalasia, Hirschsprung’s disease (HD), and diabetic colonic dysfunction [57]. Moreover, the involvement of the nitrergic system in diabetic gastropathy is related to a reduction in the active forms of neuronal NOS at the antral level, especially in women [58]. While in the jejunum, ileum, and colon of diabetic rats there is a decrease in the total number of myenteric and nitrergic neurons, in the duodenum the loss of nitrergic neurons is not accompanied by deficits in myenteric neurons [59].

Another protein involved in inhibitory enteric neurotransmission is the cyclic guanidine monophosphate (cGMP)-dependent kinase Prkg1, which is expressed by intestinal ICCs in its β isoform (PKG1β). Its modulation in NO neurotransmission has been found to be essential for ICC survival and gastrointestinal motility function [60]. ICCs conspicuously expressing the Pkrg1 protein seem to indicate that they are the main target of NO released from enteric neurons; they may also play a role in restricting nitrergic signaling at the intestinal level by intercepting NO released from the neurons closer to them. Thus, the reduction in intestinal motility due to the absence of ICCs could be explained as the consequence of excess NO at the smooth muscle level. This implies that gastrointestinal motility disorders resulting from a depletion of ICCs are also due to defects in enteric neurotransmission [60]. Another previous study of intestinal samples from guinea pigs also indicated the proximity of ICCs to enteric neurons expressing nNOS, especially at the level of ICC-IM in the circular and longitudinal layers [61].

From this, NO was concluded to be an important factor for ICC-mediated GI motility and ICC survival at physiological levels. On the other hand, the results from Kaji et al. [36] suggest that, under inflammatory conditions, an increase in NO produced by iNOS has deleterious effects on ICCs. NO-induced oxidative stress may be the main factor inducing structural changes in ICCs. Therefore, in this setting, buffering the oxidative stress is likely to prevent ICC dysfunction in patients with intestinal inflammation [53,62].

The antioxidant enzyme HO is a catalyst of the metabolism of the heme group to iron, biliverdin, and CO. It occurs in two isoforms: HO-1, the inducible form, which occurs at a low concentration in mammalian tissues; and HO-2, the constitutive isoform, which is most predominant of the CNS [63]. These enzymes are involved in heme degradation for bilirubin synthesis; it is remarkable that this catabolite is an antioxidant and neuroprotective molecule even at nanomolar concentrations [64]. In both porcine and human gastric fundus samples, the presence of HO-2 and biliverdin reductase (BVR) was evidenced by immuno-histochemistry in the gastric epithelial mucosa, the endothelium of intramural vessels, and in the myenteric and submucosal plexuses, as well as in ICCs [65]. Induction of HO protects ICCs from the increased oxidative stress associated with diabetes [62].

In a recent study of 1342 patients with type 2 diabetes, those suffering from peripheral neuropathy had lower concentrations of physiological serum bilirubin compared to controls and without neuropathy [66]. In addition, the HO/BVR enzyme system has been related to AD; alterations in the activity of these enzymes induced by oxidative stress have been described in patients with moderate cognitive impairment in the context of AD [67].

Though HO-1 induction has neuroprotective functions in inflammation, its overexpression is associated with neurodegenerative damage, particularly in diseases such as PD and AD [68]. In AD, HO-1 overactivity in astrocytes appears to amplify free radical-mediated mitochondrial damage due to iron deposition and attendant neuronal dysfunction [69]. In this regard, increased HO-1 immunoreactivity has been found in the brains of AD patients, mainly in the hippocampus and cerebral cortex, which is not evident in subjects without dementia [68].

Another antioxidant enzyme that has been studied is SOD, which is responsible for converting the superoxide free radical (•O_2_^–^) into oxygen (O_2_) and hydrogen peroxide (H_2_O_2_). Two SOD families have been identified in mammals: cytoplasmic SOD_1_, which is dependent on copper and zinc as cofactors (Cu/ZnSOD); and mitochondrial SOD_2_, which is dependent on manganese (MnSOD) [70,71]. In a study of animal samples with induced ulcerative colitis, SOD administration reduced inflammation by decreasing reactive oxygen species accumulation [72]. In dysbiosis in the nematode *Caenorhabditis elegans* exposed to the pathogen *Pseudomonas aeruginosa*, oxidative stress caused by this bacterium was found to induce SOD_1_ expression in the nervous system and intestine; deficiency of this enzyme was associated with increased lethality [73]. The high level of MnSOD labeling in parietal cells, neurons, and ICCs of the gastrointestinal tract parallels the abundance of their mitochondria, emphasizing its neuroprotective ability in enteric nerves [74].

## 4. Gastrointestinal Disorders Involving ICCs and the ENS

### 4.1. Hirschsprung’s Disease

HD is a congenital disorder consisting of an absence of ganglion cells in the Auerbach’s and Meissner’s plexuses of the distal colon [2]; this disorder is either due to an abnormal migration of the primitive cells of the neural crest between the fourth and twelfth week of gestation [2] or a degenerative process of ganglion cells by necrosis, abnormal differentiation, or changes in the cellular microenvironment [2,75]. This results in an aganglionic colon segment with absent motility and symptoms of bowel obstruction [3]. Aganglionosis may influence the proper development of intestinal pacemaker cells [76]. The most common genetic cause of HD has been associated with *RET* proto-oncogene mutation [2].

Histopathological examination of the affected colon is of great diagnostic interest because the presence of ganglion cells under the microscope rules out HD, whereas the absence of such ganglion cells together with positive staining for acetylcholinesterase in mucosal and submucosal nerve fibers diagnoses HD [2].

Studies on ICCs in these aganglionic areas have yielded contradictory results. Some studies have shown a lower cell density in affected areas, especially at the innermost levels of the circular muscle layer and in ICC-SM [20]. There are also reductions in the numbers of ICCs at the myenteric level together with an abnormal decrease in their branching between nerve trunks [16]. Other studies have shown this reduction in both resected aganglionic and ganglionic sections, suggesting that the persistence of motility problems after resection of the affected area originates in the abnormal organization of the ICCs due to either a reduction in their number or defects in their connections with the myenteric ganglia [16,20]. In immuno-histochemistry of post-operative samples from patients diagnosed with HD, the expression of connexin 43, a component of the transmembrane channels in the gap junction that connect ICCs to other structures, was decreased in aganglionic segments, favoring the lack of communication between ICCs and smooth muscle cells as being responsible for motility dysfunction [77]. There is a more severe and rare variant of HD, total colonic aganglionosis, in which c-kit-dependent immuno-histochemical alterations have also been detected, with reductions in the density of myenteric ICC networks [16].

### 4.2. Achalasia

Achalasia is described as a motor disorder of the esophagus, encompassing an absence of peristalsis together with hypertonicity of the lower esophageal sphincter, making it unable to relax [10,20]. The main characteristic of its physio-pathogenesis consists of degeneration of the ganglion cells and nerve structures of the myenteric plexus over time [10], especially in the more distal areas and lower esophageal sphincter [20]. An inflammatory etiology based on an immune reaction, perhaps due to the herpes virus or an autoimmune phenomenon with antibodies directed to the Auerbach’s plexus, has been suggested [20] as a T-lymphocyte infiltrate has been observed along the affected nerve plexuses [10]. A relationship of achalasia with paraneoplastic processes has also been proposed [19].

The damage to esophageal ICC-IM does not appear to be continuous, but patchy; this may be related to the intermembrane connection between these ICCs and the mast cells present in the muscle layer [10]. It seems that the key site of damage to these structures is located in the connections between the ICCs and the neurons, which could be responsible for the alterations in motility [22], possibly due to a defect in nitrergic neurotransmission [52]. Therefore, damage to ICCs translates ultra-structurally to a loss of connections between ICCs and neurons, a reduction in mitochondria [20], a clearer and more uncluttered cytoplasm, and sparser smooth endoplasmic reticulum [22].

Notably, in the autosomal recessive genetic syndrome Allgrove, which presents with achalasia, addisonianism, and alacrimia, there is evidence of a decrease in ICCs at the level of the gastric cardia [19,20,22].

### 4.3. Hypertrophic Pyloric Stenosis

Hypertrophic pyloric stenosis (HPS) is a pediatric disease that presents with a clinical picture of post-prandial, non-bilious projectile vomiting together with a palpable “olive” at the level of the pyloric sphincter due to pyloric sphincter hypertrophy and an inability to coordinate with gastric contractions [20]. At this level, decreases in NO-producing neurons and ICCs at the level of the hypertrophic circular muscle layer have been described in both immuno-histochemical and electron microscopy morphological studies [20]. Lack of ICCs is associated with reduced anthropyloric co-ordination as slow wave generation is compromised [22].

Pylorus samples from 18 children with HPS were found to have a reduced number of ICCs at the level of the muscular layer of the hypertrophic pylorus relative to healthy controls and a decrease in anti-HO-2 immunoreactivity, which was interpreted as a decrease in CO [29]. After surgical treatment of HPS by pyloromyotomy, the set of ICCs return to their normal pattern [20].

### 4.4. Gastroesophageal Reflux

Gastroesophageal reflux (GER) is caused by transient relaxation of the lower esophageal sphincter [22]. Sphincter control in response to increased pressure in the stomach depends on vagal reflexes [78]. The presence of gastric contents in the esophagus produces esophagitis, which can lead to loss and injury of the ICCs, especially in the more advanced stages of reflux [22]. There seems to be a direct relationship between the severity of esophagitis and ICC destruction, which causes motility problems and reflux to be maintained, exacerbating GER symptoms [19,22].

### 4.5. Inflammatory Bowel Disease

IBD includes ulcerative colitis (UC) and Crohn’s disease (CD). At the microscopic level, structural abnormalities in the ICCs have been evidenced in both UC and CD, with accumulation of lysosomes and lipid vacuoles, which could represent damage secondary to inflammatory changes [20].

The relationship between ENS and IBD remains controversial. In some cases of IBD, an abundance of enteric neurons has been seen in the inflamed tracts, raising the question of whether this enteric hyperinnervation pre-exists in genetically pre-disposed patients or whether it is a consequence of cytokine-mediated inflammatory damage [79]. Rodent studies have found an increase in macrophages in the submucosal plexus [10], suggesting that the cytokines they produce are involved in structural and functional alterations in ICCs, nerves and myocytes [20]. Nevertheless, despite such structural changes, samples from IBD patients have stable c-kit labeling, possibly due to the presence of mast cells, which label positive for c-kit and form intermembrane interactions with the altered ICCs [10]. In an experimental rodent model in which an IBD-like state was induced by administration of trinitrobenzene sulfonic acid (TNBS) and dextran sulfate sodium (DSS), enteric neuronal hyperplasia was found to be a marker of severity in IBD [79].

In conclusion, reductions in the ICCs have been seen in CD in the affected areas of the small intestine, especially the circular and longitudinal muscle layer, with some changes occurring at the level of Auerbach’s plexus and ICC-DMP [20]. The involvement of the myenteric plexus is still under discussion because KIT immunoreactivity is reduced in ICC-IM and ICC-MP from small intestine samples [10]. In UC, there are increases in KIT+ immunoreactivity at the level of the ICC-IM, with mast cells found near ICC-SMP and nerve endings [10].

### 4.6. Chagas Disease

Chagas disease is caused by the protozoan *Trypanosoma cruzi* and chronic infection can cause problems visceromegaly at both the cardiac and digestive level [80]. In the digestive tract, infection can lead to degeneration of enteric neurons and cause colorectal motility disorders, including megacolon [78]. Reductions in colon ICC density have been reported [20].

There seems to be a relationship between the chagasic megacolon and serotonin levels [7]. When comparing intestinal samples from 24 Chagas disease patients to 14 controls, patients exhibiting megacolon presented a higher concentration of mast cells and lower serotonergic expression compared to those without megacolon, who had a marked increase in serotonin [81]. Another study, which involved esophageal biopsies from 10 Chagas disease patients and 5 controls and compared the number of ICCs in the myenteric plexus, found that patients had a reduced number of ICCs in the muscle layers, which was thought to contribute to the pathogenesis of megaesophagus [80].

### 4.7. Gastrointestinal Stromal Tumors

GISTs are the most common GI tract tumors of mesenchymal origin. The majority originate in the stomach (60–70%), though 20–30% originate in the small intestine and ~10% in the esophagus, colon, and rectum [22]. They exhibit KIT and CD34 immunoreactivity, common markers of ICCs, suggesting that GISTs derive from ICCs [22]. The treatment for inoperable and metastatic GISTs is imatinib mesylate, a tyrosine kinase inhibitor targeting mutant KIT and PDGFRα isoforms [22,82].

An immuno-histochemical study found that the marker CD34 appears to be associated with GIST malignancy [25]. The fact that benign GISTs do not express CD34 could indicate that they are composed of more mature ICCs, whereas malignant GISTs may be derived from less differentiated ICCs [25]. However, another study on intestinal samples from 10 patients with GISTs to compare mutations in both the c-kit and PDGFRα genes in tumor cells with respect to ICCs adjacent to the cells found no mutations in these genes [82]. This is contrary to the theory that GISTs have their origin in ICCs or could indicate that these mutations are not germline, but acquired locally by GIST precursor cells, which contribute to tumor development along with other genetic factors [82].

### 4.8. Gastroparesia

Slow wave genesis is altered in gastroparesis, as well as in other types of functional dyspepsia [20]. In the examination of stomach samples affected by severe gastroparesis, a reduction has been demonstrated in the number of ICCs in the myenteric and intramuscular plexuses, together with hypoganglionosis and neuronal dysplasia [20]. Similar ICC findings were reported in jejunal biopsies and the *muscularis propria* of the colon from diabetic gastroenteropathy patients [20].

In gastric samples from patients with type 1 diabetes, a loss of ICC density was reported in 60 to 100% of cases [22]. Diabetic neuropathy damage extends to the vagus nerve, NO-producing neurons, ICCs, and smooth muscle [22]. The complex outcome involving ICC dysfunction as a result of alterations in the expression of their chloride–calcium channels, or even their loss, may underlie the observed delayed gastric emptying and aberrant peristalsis [11,21]. A relationship between gastric viral infections and some cases of gastroparesis has also been proposed on account of local harmful inflammation [19].

### 4.9. Post-Operative Paralytic Ileus

Post-operative ileus is not a disease in itself but a temporary reaction of the gastrointestinal system to an external aggression as direct as surgery. The motility defect has been related to an overproduction of NO at the enteric level [51]. Immediately after surgery, inhibitory neuronal mechanisms are activated, followed by another phase of infiltration of inflammatory cells, such as monocytes, neutrophils, and mast cells, into the *muscularis mucosae* [10]. However, the involvement of mast cells in pathogenesis is under discussion because no motility problems have been observed in samples from mice without mast cells but with a normal ICC network, though problems did occur in groups of ICC-deficient mice [9]. Another curious relationship between the ENS and post-operative ileus was evidenced by pharmacological stimulation of the vagus nerve at the central level, improving paralytic ileus in both the recovery of GI motility and its anti-inflammatory actions [83].

On the other hand, surgical trauma and the inflammatory cascade it triggers may influence the pacemaker activity of ICCs, temporarily decreasing it [20]. A study investigating this relationship found that inflammation appears to be mediated by NO which, in turn, causes alterations in ICCs at both the electrophysiological level, with a decrease in pacemaker activity and reduced generation and coordination of slow waves, and structural level, with increased cytoplasmic vacuolization [9].

### 4.10. Constipation

As the structure and function of organs change over the years, these changes are reflected in an increased prevalence of certain diseases associated with aging; gastrointestinal tract conditions, such as GER, irritable bowel syndrome, constipation, and fecal incontinence, are more prevalent with age [84]. There appears to be a decrease in gastric emptying and colon motility; however, the magnitude of this change remains unclear as most patients maintain relatively good function [85], though the results are conflicting [86]. Gastrointestinal motility requires normal ICC distribution and function, which is profoundly changed by ICC depletion [87] or abnormal secretion of gut neurotransmitters. Among these, NO has turned out to be the most important, though other neurotransmitters are involved as co-transmitters, such as 5-HT or vasointestinal peptide (VIP) [88]. In 2011, Gomez-Pinilla et al. [86] carried out a study aiming to clarify the effect that age has on the number of ICCs and the volume of the networks they form in both the human stomach and colon. Identifying the initial cause of ICC-related motility disorders is difficult because early damage can decrease the volume of the networks, but not by enough to cause dysfunction. Therefore, in older patients, gastrointestinal motility problems could be more common not only because the lesions on the ICC networks have become evident with age, but also because they do not possess the functional reserve that can compensate for increased aggression [86].

## 5. Neurodegenerative Diseases with Involvement of the ENS

### 5.1. Parkinson’s Disease

PD is one of the most frequent neurodegenerative pathologies in the human population. The main pathogenic phenomenon is the loss of dopaminergic neurons due to atrophy of the nigrostriatal substance, which causes bradykinesia, rigidity, and resting tremor, followed by the development of gait disturbances [89]. Elevated oxidative stress in early-stage PD patients, prior to significant neuron loss, is a robust feature. Consequently, uncontrolled reactive oxygen species generation is a potential causative factor in dopaminergic neuron death, rather than being a secondary response to progressive neurodegeneration [90].

Braak et al. [91] studied brain autopsies of patients with varying degrees of PD to infer an order of disease progression. Lesions, in the form of clumps of Lewy bodies and filaments referred to as Lewy neurites, appeared to occur initially in the dorsal motor nucleus of the vagus and glossopharyngeal nerves, eventually involving the basal ganglia and, later, the olfactory nerve and cortical areas; this order of progression appeared to progress according to disease stage, especially in the sub-group of patients who had earlier debut and faster disease progression [92]. This view is controversial, as the proposed order is not always fulfilled. The dorsal vagus nerve does not present pathological lesions in some patients with PD, while the described craniosacral distribution of α-synuclein accumulations is not always present [93]. This bidirectional relationship between PD and the vagus nerve has also been studied in vagotomized patients, who have a lower risk of PD [94]. However, this was questioned more recently in another review analyzing larger cohort studies [95].

In PD, gastrointestinal motility involvement is characteristic in all stages and often precedes the central manifestations. As pathogenic transmission from the ENS to the CNS through the vagus nerve is possible [95], this disease has been proposed to be of enteric origin [96]. In contrast, other views contended the opposite—development from the CNS to the ENS [89]. Upon inoculation of the *substantia nigra* with the neurotoxin 6-OHDA, dopaminergic neurons become damaged or reduced, whereas the number of intestinal vasoactive peptide (VIP)-producing neurons in the myenteric plexus of the proximal colon and distal ileum increased, resulting in lower peristaltic colon activity [97].

Regardless of the starting point, the pathogenesis of PD is not limited to the central nerves as originally suggested, but can be understood as a pathological disorder of the autonomic nervous system, which includes the ENS [98]. Interestingly, constipation, one of the most frequent digestive tract symptoms in PD, precedes the central symptomatology by 15 years [95].

The involvement of the ENS in the pathophysiology of PD was confirmed by the appearance of Lewy body and α-synuclein inclusions in neurons of the dorsal motor nucleus of the vagus and myenteric plexuses in duodenum and ileum biopsies from PD patients [96]. However, neither the loss of myenteric ganglia nor reductions in neuronal density in the myenteric plexus were evidenced [99]. Colonic biopsies from 35 PD patients also failed to show loss of dopaminergic and noradrenergic neurons in the submucosal plexus [100].

### 5.2. Alzheimer’s Disease

Studies of the relationship between the ENS and AD are in their early stages. This pathology is characterized by the progressive accumulation of beta-amyloid plaques, intracellular and neurofibrillary abnormal increases in *tau* protein, and central cholinergic dysfunction, which causes neurodegeneration, memory loss, and dementia [1].

As in PD, oxidative stress plays an important role in the pathogenesis of AD [84]. Overproduction of reactive oxygen species and mitochondrial dysfunction, eventually related to local calcium excess, promote *tau* protein accumulation and neurotoxicity, enhance the expression of proinflammatory cytokines (e.g., IL-1, IL-6, and TNFα), and lead to abnormal neuronal plasticity, amyloid angiopathy, vascular dysfunction, and reduced cerebral flow [101].

Importantly, amyloid precursor protein (APP), from which beta-amyloid is derived, is normally expressed in the ENS [1]. In animal studies, beta-amyloid accumulation is associated with a reduction in enteric neurons as they appear to be vulnerable to APP [1]. An immuno-histochemical study in transgenic mice with progressive accumulation of cerebral beta-amyloid described the appearance of plaques in the ganglia of enteric neurons and linked APP overexpression to the loss of neuronal structures in the ENS, motility disturbances, and smooth muscle atrophy [102].

Nevertheless, the enteric extension of AD pathology is variable. In mice with the Thy-APP23 mutation of APP, motor neuronal groups, both excitatory substance P-dependent and inhibitory NO-dependent, did not seem to be affected even though amyloid deposits were evidenced in the intestinal lamina propria, favoring clear involvement of the ENS in the neurodegenerative process of AD [103]. In contrast, human intestinal samples, though in a small number, demonstrated a submucosal increase in beta-amyloid in AD patients [1] and a good correlation between brain and intestinal beta-amyloid and APP levels [104].

### 5.3. Amyotrophic Lateral Sclerosis

ALS is a progressive neurodegenerative disease that primarily affects the motor neurons of the medullary anterior horn, as well as the motor nuclei and motor cortex of the brain [105]. Patients present with a high prevalence of gastrointestinal symptoms, such as dysphagia, slowed gastric emptying, and colonic transit dysfunctions [96].

Mutations in the gene encoding of the nuclear ribonucleoprotein TDP-43 have been identified in some forms of familial ALS and frontotemporal dementia [106]. One consequence observed in transgenic mice is the appearance of post-translational changes, including abnormal cleavage, hyperphosphorylation, and ubiquitin tagging, which result in impairment of protein nuclear localization, cytoplasmic accumulation, and neurodegeneration [107,108]. Other genes implicated in ALS are *SOD* enzyme genes encoding and *FUS,* since their mutations lead to RNA dysregulation, resulting in accumulation of intraneuronal aggregates and defective axonal transport [109].

In a murine experimental study in which the mutated SOD protein from human spinal cord was inoculated in the spinal cords of mice, its accumulation in the form of aggregates and distribution along the neuroaxis was demonstrated, leading to progressive neuronal loss and paralysis; in this way the ALS disease was transmitted [110]. Up to 150 *SOD1* mutations have been described, resulting in increased production of oxygen free radicals [111]. In addition, mutated *SOD1* can directly interfere with the functioning of neuronal mitochondria, especially at the synaptic level [106], and lead to genome abnormalities [101].

FUS and TDP-43 both contain domains rich in glutamine and asparagine residues [112], which relate to the inhibitory circuits of cellular autophagy [109]. Moreover, dysfunction of these autophagy circuits, leading to accumulation of these proteins, is a mechanism that also seems to be shared by other neurodegenerative diseases with accumulations of other proteins, such as α-synuclein in PD or beta-amyloid in AD [109]. The neuronal injury may start in the ENS, from where it spreads to the CNS as retrograde neurodegeneration, a behavior comparable to the way prion diseases spread [1,109].

Importantly, motor neurons affected in ALS have higher energy requirements. Thus, mitochondrial dysfunction related to oxidative stress is more accentuated, precipitating chronic neuroinflammation [105] and aberrant activation of microglial cells and astrocytes [102]. Therefore, ENS dysfunction triggered by inflammation and oxidative stress could be involved in the pathogenesis of ALS.

Samples from the intestines of ALS mice presented an increase in abnormal Paneth cells, as well as pro-inflammatory cytokines, such as IL-17, in both the blood and intestinal tissues [96].

Neurological diseases, such as PD, AD, ALS, and multiple sclerosis [113], are often associated with functional gastrointestinal disorders, which may occur at all stages of disease progression, to such an extent that these digestive and intestinal alterations are now considered an integral part of their clinical picture. Gastrointestinal dysfunction due to changes in the gut microbiota and enteric neuroimmune system alterations could contribute to the initiation and upward spreading of the neurological disorder in central neurodegenerative diseases.

## 6. Neurodevelopmental Disorders with Involvement of the ENS

### 6.1. Autism Spectrum Disorders

Autism is a neurodevelopmental disorder diagnosed in children whose most frequent signs are social withdrawal, communication problems, and repetitive behaviors [1]. A meta-analysis of 15 previous studies designed to compare the gastrointestinal symptoms of children with autism with those of controls concluded that children with autism have a higher incidence of diarrhea, constipation, and abdominal pain [114]. A subsequent meta-analysis of 22 studies comparing the blood level of serotonin (5-HT) in autistic patients with respect to controls found that patients with ASD have higher blood levels of 5-HT [115]. The fact that 5-HT increases correlate with ASD may indicate the influence of ENS as the highest concentration of blood 5-HT is of intestinal origin [1]. Marler et al. reported that 23% of children and adolescents with ASD have hyperserotonemia; functional constipation was also frequent in these children and adolescents, suggesting a relationship between serotonin levels in blood and the appearance of digestive symptoms [115].

Mutations shared by ASD and gastrointestinal motility disorders include *CDH8*, *MET*, *TCF4*, and sodium-dependent 5-HT transporters SERT/SLC6A4 [3]. The mutations that affect neuronal communication could result in gut dysfunction and lead to disruption of the intestinal microbiota. An increased number of small intestine myenteric neurons have been found in patients and mice with *neuroligin-3 R451C* mutation. As this finding indicates gastrointestinal alteration, this mutation has been proposed as a pre-clinical model for the study of ENS dysfunction in autism [116]. In this model, cecal ganglia contain more neurons overall and increased numbers of NO neurons per ganglion in both the myenteric and submucosal plexuses [117].

### 6.2. Attention-Deficit/Hyperactivity Disorders

The roots and consequences of autonomic imbalance on the symptomatology of this disorder remain largely unproblematized in the literature, while few studies have addressed the role of the third autonomic branch, i.e., the ENS [118].

The pre-natal and early post-natal stages represent a critical time window for human brain development. Interestingly, this window partly overlaps with the maturation of the intestinal flora (microbiota) that play a critical role in the bidirectional communication between the CNS and ENS (microbiota–gut–brain axis). The microbial composition has important influences on general health and the development of several organ systems, such as the gastrointestinal tract, immune system, and brain. Clinical studies have shown that microbiota alterations are associated with a wide range of neuropsychiatric disorders, including ASD, attention deficit hyperactivity disorder, schizophrenia, and bipolar disorder [119].

Borderline cognitive abilities, attention-deficit disorders, and possible epileptic seizures were noticed in patients with HD. These patients remain a clinical and functional challenge in spite of the availability of management guidelines [120].

### 6.3. Down Syndrome

Trisomy 21 affects the developmental processes of the nervous system, including neural patterning, neuronal and axonal migration, and synaptic development. In addition to factors influencing neural development, there is the critical issue of timing as the ENS develops in a tight spatiotemporal relationship [121]. The existing dendritic spines appear abnormally long, thin, or irregular in contour and appearance [122].

In Down syndrome, there is a reduced number of neurons in the esophageal plexus ganglia [123]. The high frequency of bowel problems in children with Down syndrome and HD may occur because of additional unrecognized problems with the ENS structure. In two mouse models of Down syndrome, i.e., *Ts65Dn* and *Tc1*, the submucosal plexus neuron density was markedly reduced throughout the bowel [124].

## 7. Conclusions

ICCs are localized in the gut and at the heart of enteric neurotransmission. Acting as the main pacemaker cells of the ENS, they are responsible for the genesis of slow waves and the gut response upon mechanoreceptor sensing. In the main gastrointestinal pathologies, there is evidence of motility disturbance or failure in myenteric plexus and ICC connections.

Apart from their distinctive neurological signs, neurodegenerative diseases, such as PD, AD, and ALS, and neurodevelopmental disorders, such as ASD, exhibit a variety of signs of gastrointestinal dysfunction. The characteristic accumulation of abnormal proteins at the cerebral level, such as Lewy bodies in PD or β-amyloid in AD, is now recognized to extend to the enteric nerve plexuses. In ALS, further research is needed to clarify whether the accumulation of TDP-43 aggregates in the CNS could reach the gut or whether, by retrograde neurodegeneration, the injury originates in the ENS. Although several findings point to a bidirectional relationship between the CNS and ENS in the context of disease, further studies are needed to clarify the mechanisms that explain this relationship.

An imbalance between pro-oxidant and antioxidant mechanisms, including a reduction in nitrergic pathways, overexpression of HO-1 in neuronal tissues, and mutations in SOD functioning may impact on ENS and ICCs functions.

## Data Availability

Not applicable.
